# Multivariate analysis for FTIR in understanding treatment of used cooking oil using activated carbon prepared from olive stone

**DOI:** 10.1371/journal.pone.0232997

**Published:** 2020-05-22

**Authors:** Sara M. Alshuiael, Mohammad A. Al-Ghouti

**Affiliations:** Department of Biological and Environmental Sciences, College of Arts and Sciences, Qatar University, Doha, State of Qatar; Luleå University of Technology, SWEDEN

## Abstract

In this study, activated carbons prepared from the green and black olive stone (green OSAC and black OSAC) were used as adsorbents to investigate their removal efficiencies for oxidation products and polar compounds from used sunflower and corn cooking oils. The degree of oxidation level and polar compounds were evaluated using Fourier transform infrared (FTIR) with the principal component analysis and ultra-performance liquid chromatography. Two FTIR absorption peaks were used for the oil evaluation, namely 3007–3009 cm^-1^, which is related to C-H symmetric stretching vibration of the *cis* double bonds, and ~1743 cm^-1^, which is related to = CH and ester carbonyl stretching vibration of the functional groups of the triglycerides, C = O. The principal component analysis results showed significant variations in the oxidation level of the sunflower and the corn oils occurred after consecutive heating and French fries frying for 10 days. The oxidation products that are adsorbed on the surface of the OSAC forms π-complexes with the C = C parts of the OSAC system. It can be concluded that the prepared adsorbents can be promising, efficient, economically effective, and environmentally friendly alternative adsorbents for oil treatment applications.

## 1. Introduction

With the increased development in industry and economy, various environmental and health issues resulted from climate change. To reduce the effects on climate change, various actions and solutions should be taken. Among these, olive and olive oil production as one of the most widespread agricultural activities throughout the Mediterranean region plays an important role in protecting the environment by mitigating the greenhouse effect. Olive trees act as a safeguard for the biodiversity and improve the soil. A quantifiable amount of carbon is stocked in an olive grove and consequently CO_2-eq_ [1]. In 2005/2006, olive and olive oil productions around the world were estimated to be 1.73 and 2.58 million tons/year [2]. While in 2010, olive oil production was around 2,881,500 metric tons globally, and between 1990 and 2010, it was estimated that the worldwide olive oil consumption increased by 78% [3,4]. Consequently, producing large amounts of olive oil causes the production of large amounts of solid waste in the form of olive stone. These by-product waste residue cannot be naturally degraded easily, and their disposal is a major environmental problem for leading countries in olive oil production. Olive stone mainly constitutes of cellulose, hemicellulose, and lignin as the major components with a ratio of 16.6–37%, 14.4–36.6%, and 20–43%, respectively. Ash content is around 5%, moisture contents of olive stone range between 40 to 75%, depending on the extraction process for the production of oil. Protein content is approximately in the range of 5 to 8%, and fat content between 3.5 and 18% depending on the extraction process. Furthermore, carbon, hydrogen, nitrogen, oxygen and sulfur were in the range of 46.6–57.8, 6.07–9.2, 0.6–1.0, 34.4–37.3, and 0.11–0.13%, respectively [5].

For many years, the treatment of waste cooking oil like sunflower oil, corn oil, and others is a major environmental concern [6,7]. The frying process is one of the most commonly used cooking methods in the food industry, in which it causes oil oxidation in vegetable oil and a reduction in food quality because of the usage of the high temperature over long periods [8,9]. Fresh vegetable oil compounds are mainly non-polar [10]. Complex reactions of oxidation, polymerization, and hydrolysis of hydrocarbons within the oil happens during the frying process [11]. A variety of polar compounds are produced more than any other substances, which accelerate the breakdown of structural properties of the oil causing deterioration [12,13].

Volatile substances, as one of the decomposition products of cooking oil, occur at concentrations of one part per million, although they are tremendously essential to the flavor qualities. Nonvolatile polar compounds and triacylglycerol dimers and polymers are the primer products of cooking oil deterioration [12]. Related to the nonvolatile polar compounds, dimers, and polymers, the quantities of cyclic compounds are quite small. Dimers and polymers are considered enormous molecules, which is formed by a mixture of -C-C-, -C-O-C-, and -C-O-O-C- bonds [13]. Water, vapor, and oxygen are the initiators of the chemical reactions that happen in the cooking oil and food. This because when the oil is hot, and the food is introduced to it, the moisture steam evaporates with a bubbling action and progressively diminishes. Water is considered a weak nucleophile, result in di- and monoacylglycerols, glycerol, and free fatty acids, when it contravenes the ester linkage of triacylglycerols. As the number of frying rises, free fatty acid substances in the oil rise as well. To observe the properties of frying oil, free fatty acid value is used as a primary value. Thermal hydrolysis mostly occurs in the oil phase, not at the interface of water with oil. Hydrolysis of oil raises as the interaction of food moisture with oil increases. When deep-fried potatoes are fried using cottonseed oil in the temperature range between 155 to 195°C, mono- and diacylglycerols increment until reaching a plateau. To decelerate the hydrolysis of cooking oil, the frying oil should be regularly replaced with fresh oil [11–13].

Sunflower oil is one of the popular oil used in food. Sunflower seeds have a high oil content (22–36%). Sunflower oil is a triglyceride with a high content of monounsaturated acid and polyunsaturated acids. There is clear evidence that sunflower tends to polymerize when used for frying. This is true for all the edible fats and oils where they have autoxidation at low temperatures. At high temperatures, like in deep frying, they easily undergo polymer formation in a high fraction. Corn oil is also a popular cooking and frying oil, and one of the high-quality, good cooking oils, which is utilized in high amounts in houses and industries.

Chemically, the thermal degradation mechanism is comparable to autoxidation at high temperatures. However, at a higher frying temperature some reactions activated energy is exceeded, where some other reactions other than the autooxidation will be possible and other reactions pathways will occur. It should be considered that oxidation is the real adversary of oil, where the molecules of oil react with oxygen [12]. It is found that how speedy oxidation occurs will rely upon the sort of oil that is utilized. For instance, unsaturated fatty acids oxidize more rapidly than saturated fatty acids. In this way, oils with more saturated fatty acids are more oxidation stable [13].

Although various analytical methods and techniques have been reported for the determination of oxidation products they required toxic and hazardous solvents, sample preparation, and need more time of analysis [11]. Owing to the simplicity and promptness, Fourier transform infrared (FTIR) spectroscopy has been acknowledged as an effective technique to determine many parameters of fats and oils including carbonyl and polar compounds [11]. FTIR is characterized as the main tool used for monitoring processes in the food industry because of its affordability, high performance, and convenience compared to other methods. Besides, owing to the high molecular weight and the limited thermal stability of polar oxidation products, high-performance liquid chromatography (HPLC) was used to study them at different stages of heating.

To reduce the FTIR data redundancy and get proper analytical information from overlapped FTIR spectra, multivariate analysis (principal component analysis, PCA) and multivariate calibration have been used [11,14]. The PCA technique is used for the identification of a smaller number of uncorrelated variables, known as principal components (PCs), from a larger set of data that help to emphasize variation in the dataset. Trubetskaya et al., 2019 [14] investigated the yields of different tar compounds from lignocellulosic compounds based on the change in temperature and residence time. It was shown that orthogonal projections to latent structures discriminant analysis (OPLS-DA) model based on the experimental mass spectrometry data can explain the differences in tar composition. It was used to increase the interpretability of models by separating the variation that is related and unrelated to the response. Using the same settings, the prediction properties are the same for OPLS and the partial least squares (PLS).

Every day, huge amounts of different types of cooking oils are used in restaurants, industries, and households. These oils are usually thrown away as waste. The untreated waste oil pollutes the environment and it exceeds the environmental degradation capacity limit. Thus, the best way to deal with waste oil is to treat it by converting it into biodiesel, animal food, or other industrial uses like soap productions. To enhance the quality of used cooking oils, oils should be remediated using various remediating materials such as carbonaceous and inorganic adsorbents [7, 10]. Among the variety of different adsorbents is activated carbon, which takes a leading position as an adsorbent for the removal of various toxic pollutants due to its excellent adsorption ability [15–27]. Activated carbons are known to have a large surface area, polar functional groups, and microporous structure, hence, it is not effective in removing polar substances from used frying oil and does not allow efficient purification. Thus, activated carbon can be modified by oxidation to improve the surface polarity by increasing the number of functional groups with polar characteristics. Making it more effective at removing the compounds that cause deterioration in oil quality and physical characteristics. Here, activated carbon prepared from olive stone (OSAC) for the treatment of waste cooking oil was proposed. Moreover, OSAC usually are used for the removal of dyes, odors, metals [25–27]. However, based on our knowledge, there are no studies on the use of activated carbon prepared from olive stone for the treatment of waste cooking oil.

Hence, the objectives of this research were to: (i) prepare and characterize activated carbons obtained from black (black OSAC) and green (green OSAC) olive stone using a scanning electron microscope (SEM), Fourier transform infrared (FTIR), carbon, hydrogen, nitrogen, sulfur analysis (CHNS), and pH of solution (pH_solution_), surface area and pore size distribution, (ii) study the oxidation products formed during the frying process of oil using FTIR and ultra-performance liquid chromatography (UPLC), (iii) investigate the removal efficiency of the oxidation products using the prepared activated carbon adsorbents (black OSAC and green OSAC), and (iv) employ FTIR spectroscopy with multivariate analysis (PCA) to evaluate the formation of oxidation products.

## 2. Methodology

### 2.1. Sample preparation

**Fig 1:**
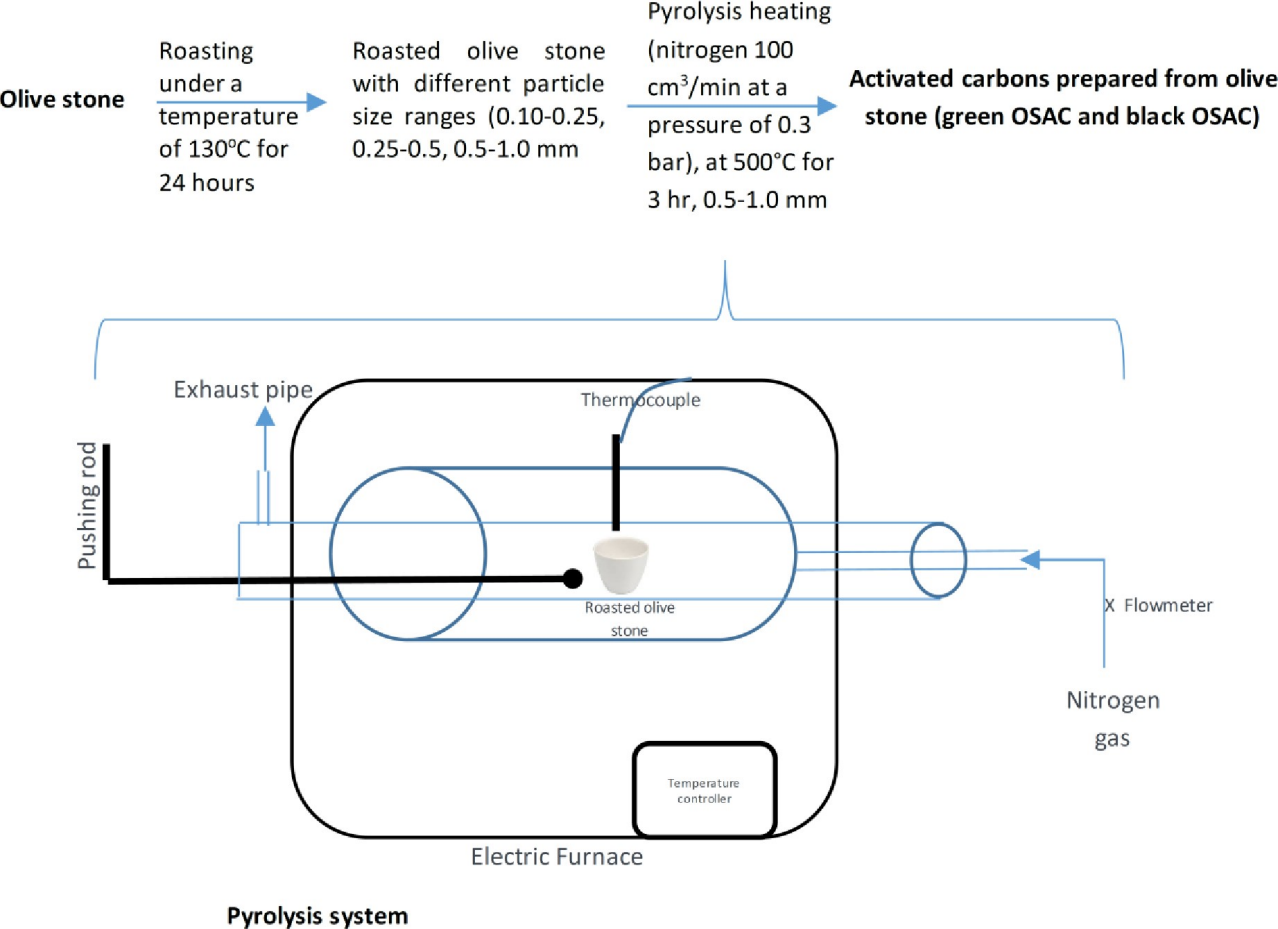
Setup of the OSAC preparation and the pyrolysis system used in the study.

Two representative samples of olive stone from black and green olives were collected. No specific permissions were required for these locations/activities. We confirm that the field studies did not involve endangered or protected species. The olive stone samples were washed thoroughly with distilled water to remove impurities and then roasted under a temperature of 130°C for 24 hours. The olive stone samples were crushed and sieved to form different particle size ranges (0.10–0.25, 0.25–0.5, 0.5–1.0 mm). The ground olive stone was stored in glass bottles. To prepare the black OSAC and green OSAC, the samples were placed in a separate ceramic crucible inside the programmable electric-heated tube furnace (Nabertherm GmbH, Germany) with a continuous flow of nitrogen 100 cm^3^/min at a pressure of 0.3 bar. The furnace was first maintained at room temperature for 50 min to make sure that the air is completely purged and replaced with the flowing nitrogen. The furnace was heated up to a temperature of 500°C with a heating rate of 10˚C/min. After that, the samples were placed in the furnace at 500°C for 3 hours and were cooled down spontaneously to room temperature while passing nitrogen gas, as shown in Fig 1.

### 2.2. Physical and chemical characterization of black OSAC and green OSAC

The black OSAC and green OSAC were characterized using SEM (JEOL model JSM-6390LV, Resolution: 3.0 nm at 30 Kv and 15 nm at 1 Kv, imaging modes: secondary electron (SE), backscattered electron (BSE), Accelerating voltage range: 0.5 Kv– 30 Kv) with 900x magnification), and FTIR (Perkin Elmer Model 2000, resolution of 1.0 cm^-1^). The FTIR measurements were performed over 4000–400 cm^−1^. The CHNS (FLASH 2000) elemental analysis and the pH_solution_ were also examined. The pH_solution_ was prepared by adding 1 g of the sample in 100 mL distilled water, then shaken for 24 hours. Bulk and particle densities of the adsorbents were also determined. For the Brunauer-Emmett-Teller (BET) surface area, pore radius and pore volume, BET model Aim Sizer-AM301 was used.

### 2.3. Heating and frying procedure

Two liters of each of fresh sunflower oil and corn oil were used throughout the experiments. The heating process was performed using a beaker (capacity 5 L) and a controlled temperature up to 180 ±10 ^o^C. This process was carried out for 1 hour daily for 10 days. Each day, an oil sample was withdrawn into 5 capped vials, 4 of them with 3 mL oil of sample and one with 1 mL oil sample. After that, the vials were stored at room temperature for further analysis. A total of 50 vials of each oil were collected. Furthermore, another 2 L of each of fresh sunflower oil and corn oil was used for the French fries frying process. At a stovetop, a stainless-steel pot was used to heat the oil to 180 ±10 ^o^C. A 250 g of frozen French fries were weighed and divided into 3 batches were added and fried one by one, until reaching consumer quality. After repeating the frying process for 10 days, the oil samples were withdrawn into 5 capped vials, 4 of them with 3 mL of oil sample and one with 1 mL oil sample and stored at room temperature for further analysis.

### 2.4. FTIR experiments

One milliliter of the oil sample was withdrawn each day after cooling to investigate the formation of oxidation products in the oil samples. This was carried out using FTIR (Perkin Elmer Model 2000). All absorbance spectra were obtained in the 4000–400 cm^-1^ range by 100 scans at 1.0 cm^-1^ resolution. A liquid cell equipped with quartz windows was used to record the spectra. A 0.025 mm spacer was used. All samples were measured in duplicate.

### 2.5. Solid-phase extraction (SPE) and the ultra performance liquid chromatography (UPLC) experiment

Massachusetts EPH SPE Cartridge was used. The column was firstly rinsed with 30 mL dichloromethane. Then, a one-milliliter sample was loaded on the SPE cartridge followed by 20 mL from dichloromethane. After that, the extracted solution was evaporated by nitrogen to get 1 mL concentrated polar compound solution of oil. The extracted sample from the solid phase extraction was examined by UPLC (Waters, Model Acquity; The ACQUITY UPLC® System, USA) using ZoRBaX RP-HPLC Column C18 (octadecyl) phase (L × I.D.: 25 cm × 4.6 mm, particle size: 5 μm separation technique: reversed phase) with 50:50 (V:V) Acetonitrile: water in gradient mode at flow rate 1 mL/min, peaks were at 254 nm with UV detector (injection volume = 10 μL, run time = 10 min, and mode of separation: gradient elution). The solvent system used had water (polarity index = 10.2) and miscible organic solvent (acetonitrile, polarity index = 5.8). The proportions of water to non-polar solvent were optimized such that the capacity factor of the last eluted analyte gets a value of nearly 2. The HPLC-grade acetonitrile was procured from Sigma-Aldrich. The quantitative analysis of the oxidation products was achieved with an external standard method.

### 2.6. Multivariate analysis

Different mathematical and statistical methods can be used to extract useful information from data of measurements, which is known as multivariate analysis. PCA is a kind of multivariate analysis that can help in providing useful interpretation regarding variance in multivariate and multispectral data set [28]. Spectroscopic techniques as they produce large amounts of data, often with multi-dimensional complications are more applicable for the data extraction. Unscrambler X (v10.5, Camo Analytics—USA) following singular value decomposition (SVD) algorithm and XLSTAT 2016 (MS Excel 2016, Microsoft–USA) was used for the PCA and clustering of variables. In this paper, PCA was applied to differentiate FTIR obtained at various experimental conditions.

### 2.7. Adsorption isotherm—batch experiments

The batch adsorption experiments were conducted in 5 mL capped vial, each with 3 mL oil (oxidized sunflower oil and corn oil obtained after the heating and frying procedures) and 0.03 g of the black OSAC or green OSAC. The samples were left in the shaker for 24 hours at 230 rpm. Then, the oil samples were filtered from the black OSAC or green OSAC. The results of each batch experiment were analyzed using UPLC and FTIR to determine the adsorption efficiency of the oxidation products onto black OSAC or green OSAC.

## 3. Results and discussion

### 3.1. Physical and chemical characterizations of the black OSAC or green OSAC

#### 3.1.1. Scanning electron microscope (SEM)

**Fig 2:**
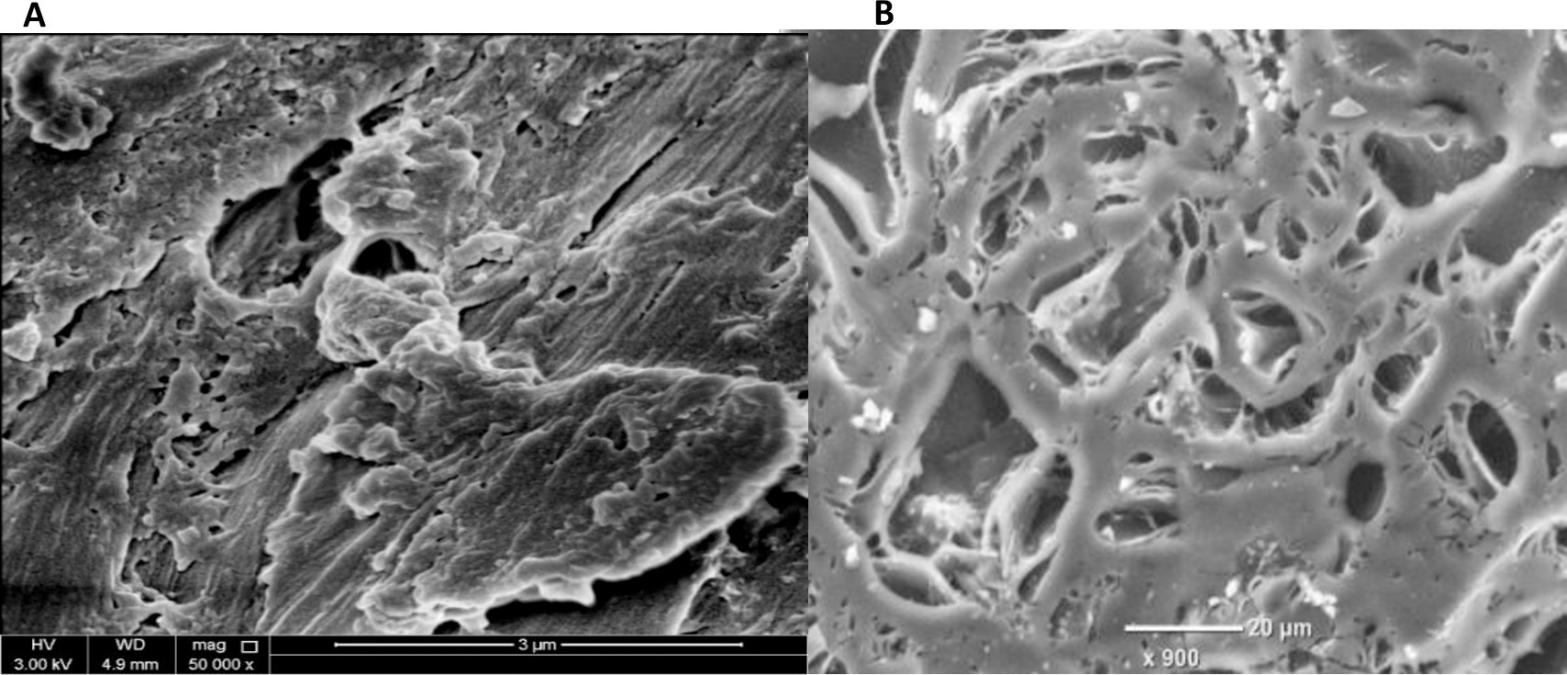
SEM image of (a) black olive stone, (b) black OSAC.

Fig 2 shows the SEM images of the black olive stone and the black OSAC. Fig 2A describes the black olive stone as large irregular crevices, macropores, mesopores and cracks, with rough edges along with thick wall structure. Fig 2B represents the black OSAC as an irregular porous structure with large, deep well-developed cavities. These macro/mesopores developed in the olive stone when it was pyrolyzed to activated carbon mainly due to the evolving of the volatile organic compound in the used raw material that was exposed to extremely high temperatures, resulting in a cracked surface [29]. The porous surface enhances the quality of the black OSAC, and the possibility of adsorption and trapping the oxidation products in the crevices [30].

### 3.1.2. Fourier transform infrared spectroscopy (FTIR)

**Fig 3:**
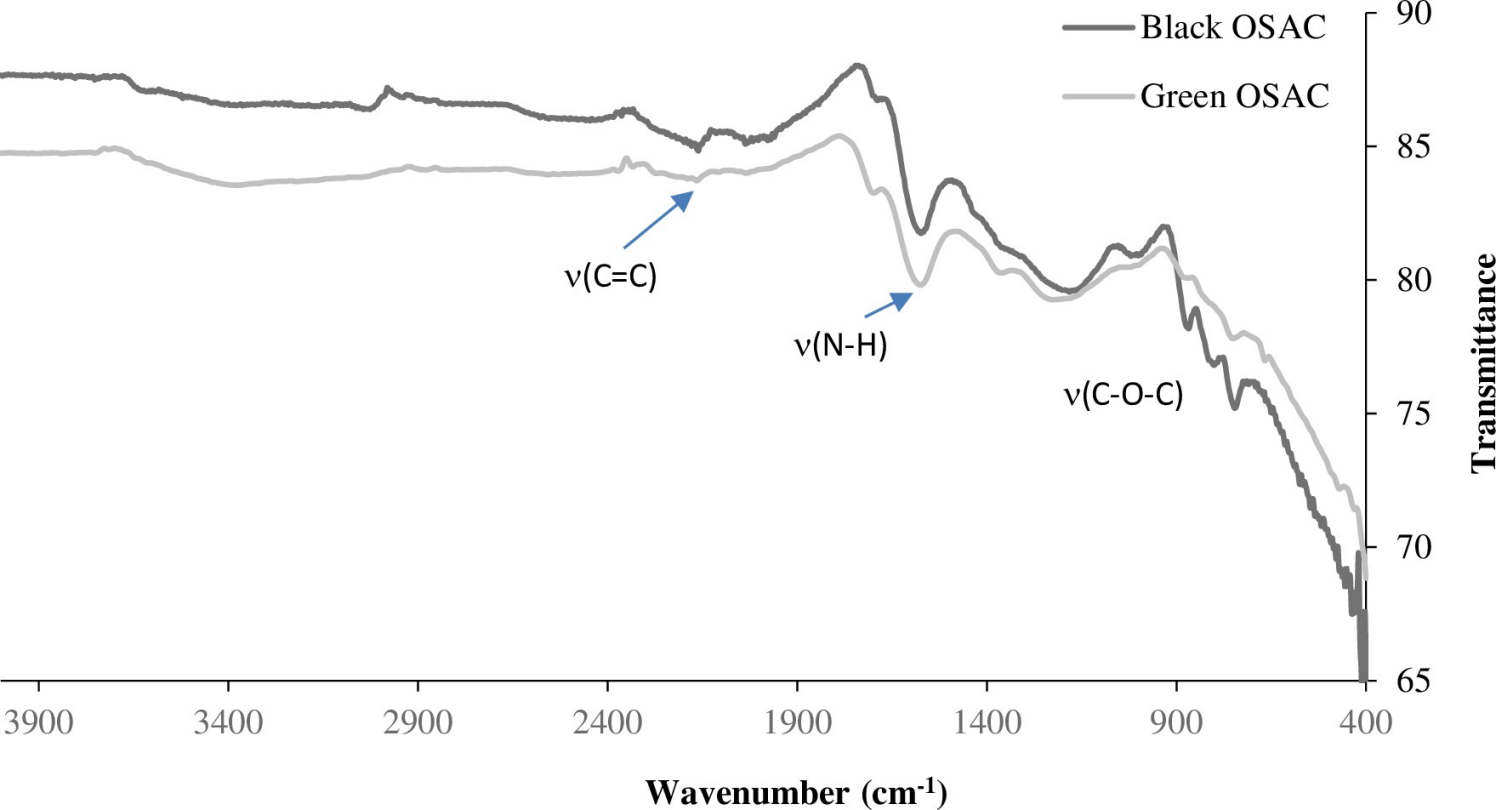
FTIR spectra of black OSAC and green OSAC.

The FTIR spectroscopy technique was used to identify the functional groups on the surface of the black OSAC and green OSAC, as shown in Fig 3. The absorption bands at 3600–3200 cm^-1^ were due to the stretching vibration of the hydroxyl group (νO–H). The absorption bands at 1600–1400 cm^-1^ (centered at 1567 cm^–1^) were due to the stretching vibration of νC = O and νC = C, or by the in-plane bending vibration of δN–H, indicating that the black OSAC and green OSAC contained some carboxylic acid, fatty ketone, or amino groups functional groups. The 2158 cm^–1^ was attributed to C = C stretching vibrations of quinoid structure [31]. The peaks at 1300–1000 cm^-1^ (centered at 1151 cm^–1^) were recognized to the νC–O stretching vibrations, which are resulted from the hydroxyl, ester, and ether functional groups on the surface of the black OSAC and green OSAC. The observed functional groups could be due to the composition of the olive stone, which is mainly composed of cellulose, hemicellulose, and lignin. The FTIR band characteristics are as follows: is N-H of amines and C = C stretching of alkene, and 1151 cm^–1^ is C–O stretching and C–N stretching of amine [32–37].

### 3.1. 3. CHN elemental analysis, pHsolution, pore size and surface area characterization, bulk density and particle density of the green OSAC and black OSAC

The results showed that the highest elemental composition of the green OSAC and black OSAC was carbon followed by hydrogen, then nitrogen, with no sulfur, and the rest was ash residue. Table 1 illustrates the obtained CHNS elemental analysis results compared to commercial AC elemental analysis [37]. In comparison with the commercial activated carbon (CAC) (C (88.0%), H (0.50%), and N (0.50%), the black OSAC displayed closer chemical composition with the commercial activated carbon more than the green OSAC. The results showed higher hydrogen content in the OSAC more than the commercial one, this can be due to the presence of water [38,39].

**Table 1:** CHN elemental analysis, pH_solution_, pore size and surface area characterization, bulk density and particle density of the green OSAC and black OSAC.

	**CHN elemental analysis**		
Green OSAC	66.3%	2.41%	0.180%
Black OSAC	84.3%	2.59%	0.260%
	**pH**_**solution**_		
	**before adsorption**	**after adsorption**	**Δ pH**
Green OSAC	7.88	7.29	-0.59
Black OSAC	7.37	7.41	0.04
	**Bulk density (g/cm^3^)**	**Particle density (g/cm^3^)**	
Green OSAC	2.0	2.4	
Black OSAC	2.0	3.3	
**Pore size and surface area characterization**			
	**Volume of the macropores** **(cm**^**3**^**/g)**	**Volume of the mesopores** **(cm**^**3**^**/g)**	**Specific surface area (S**_**BET**_ **(m**^**2**^**/g)**
Green OSAC	0.101 ±0.006	0.0501 ±0.0019	9.11 ±0.12
Black OSAC	0.094 ±0.002	0.0599 ±0.0013	9.74 ±0.22

### 3.2. Physical and chemical characterization of cooking oil

#### 3.2.1. Physical and chemical parameters of sunflower and corn oil

Table 2 shows the physical and chemical parameters and properties of the sunflower and corn oils gathered from different studies [40–46]. As shown in Table 2, sunflower oil has a higher melting point, flash point, smoke point, iodine value, saponification value and peroxide value, while corn oil has a higher fire point and viscosity at 30˚C. Furthermore, both oils have equal higher heating value and lower heating value.

**Table 2:** Physiochemical properties of sunflower oil and corn oil [40–46]. Mean ± standard deviation of triplicate determination.

Parameter	Sunflower Oil	Corn Oil
Melting point (˚C)	17.0 ± 1.15	-11 to -8
Flash point (˚C)	339.00 ± 2.20	332 to 338
Fire point (˚C)	342.00 ± 1.20	366 to 371
Smoke point (˚C)	235.00 ± 1.75	230 to 238
Turbidity point (JTU)	8.00 ± 1.10	-
Soft point (˚C)	42.00 ± 1.10	-
Cloud point (˚C)	-	-14 to -11
Acid value (%)	3.09 ± 0.42	-
Free fatty acids (%)	0.110	0.125
Free fatty acid (% oleic acid)	1.40 ± 0.07	-
Free fatty acids RBD (%) max	-	0.05
Iodine value (WIJ)	131.60 ± 0.71	127 to 133
Saponification value (mg KOH/g oil)	197.43 ± 0.42	187 to 193
Peroxide value (meq/kg)	1.0	1.0
Peroxide value (meqO_2_/ kg)	12.6 ± 2.20	-
Color Lovibond	-	3.0 red max
Color	18.00	-
Red	25.0	0.9
Yellow	0.4	25.0
Specific gravity	0.923 ± 0.016	-
Surface tension, 25˚C (dyn/cm)	-	34.80
Interfacial tension, k H_2_O at 24˚C (dyn/cm)	-	18.60
Viscosity at 30˚C	28.3	28.7
Viscosity at 40˚C	-	30.80
Viscosity at 60˚C	-	18.15
Unsaponifiable (%)	-	1 to 3
Yield (%)	41.3 ± 2.10	-
Gardner	-	6 max.
Dielectric constant 26 ˚C	-	3.954
Thermal conductivity at 130 ˚C (J/s/cm^2^/˚C)	-	4.2017 x 10^−5^
Weight per gallon at 60 ˚C (pounds)	-	7.7
Higher heating value (MJ/Kg)	39.5	39.5
Lower heating value (MJ/Kg)	36.59	36.59

### 3.2.2. Fourier transform infrared spectroscopy (FTIR) experiment

The FTIR analysis was carried out for the oil samples (sunflower and corn oils) before and after the batch adsorption experiments to compare the oxidation level before and after adsorption and determine the adsorption efficiency of the green OSAC and black OSAC.

**Fig 4:**
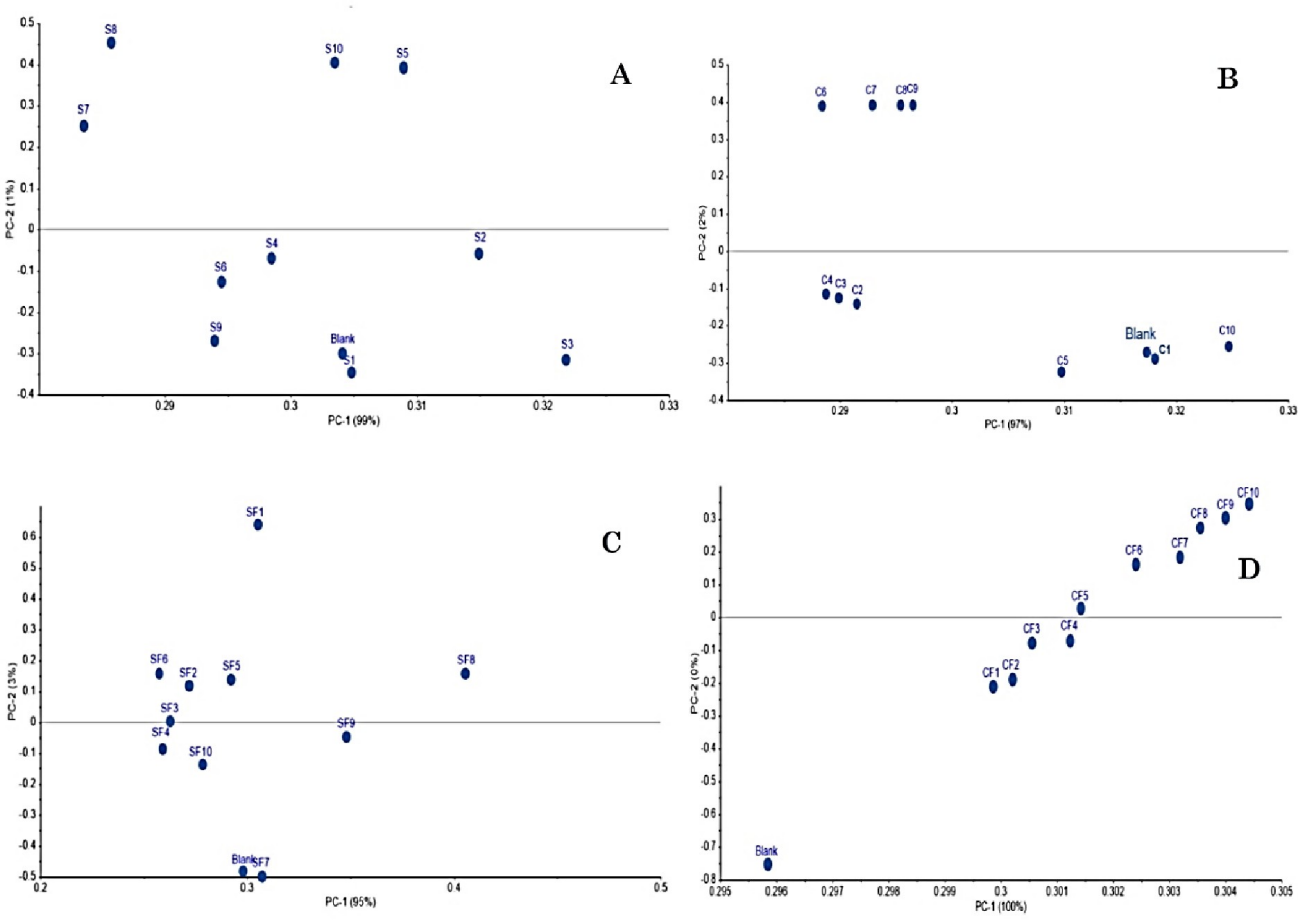
PCA plots for the obtained FTIR spectra of (a) sunflower oil (S) after heating for 10 days, (b) corn oil (C) after heating for 10 days, (c) sunflower oil after French fries frying (SF) for 10 days, (d) corn oil after French fries frying (CF) for 10 days.

**3.2.2.1. FTIR analysis–used sunflower and corn oils–before the batch adsorption.** The FTIR analysis was carried out for the sunflower and corn oils after the heating and frying processes to study the formation of oxidation products. In addition, the PCA was carried out for the FTIR spectra obtained for sunflower and corn oils after heating (Fig 4A and 4B) and after French fries frying (Fig 4C and 4D). The PCA helped in plotting the variation resulted in the FTIR spectra of oil after use (heating/frying) for 10 days. The first principle component (PC1) accounted for 99%, 99%, 97%, and 100% of the variability in the data set and the second principal component (PC2) accounted for 1%, 2%, 3%, and 0% as shown in Fig 4A, 4B, 4C, and 4D, respectively. The obtained data are in correlation with Wu and Zhao [47], who found that PC1 had more correlation with the variables than other performed PCs. It is not surprising that PC1 had more correlation with the variables than PC2, due to the fact that PCs extraction occurs successively, as each one accounts for as much of the variance remaining as possible [48]. The plots of PCA showed that after 1^st^ day of use, there was no significant change in the oil contents and therefore, the sunflower and corn oils obtained spectra from 1^st^ day samples were plotted beside the blank (unused oil). However, with the increase in use for heating or frying, the content of oil varied which caused obvious changes in the FTIR spectra. A similar trend was noted in the PCA results of FTIR spectra obtained after French fries frying.

**Fig 5:**
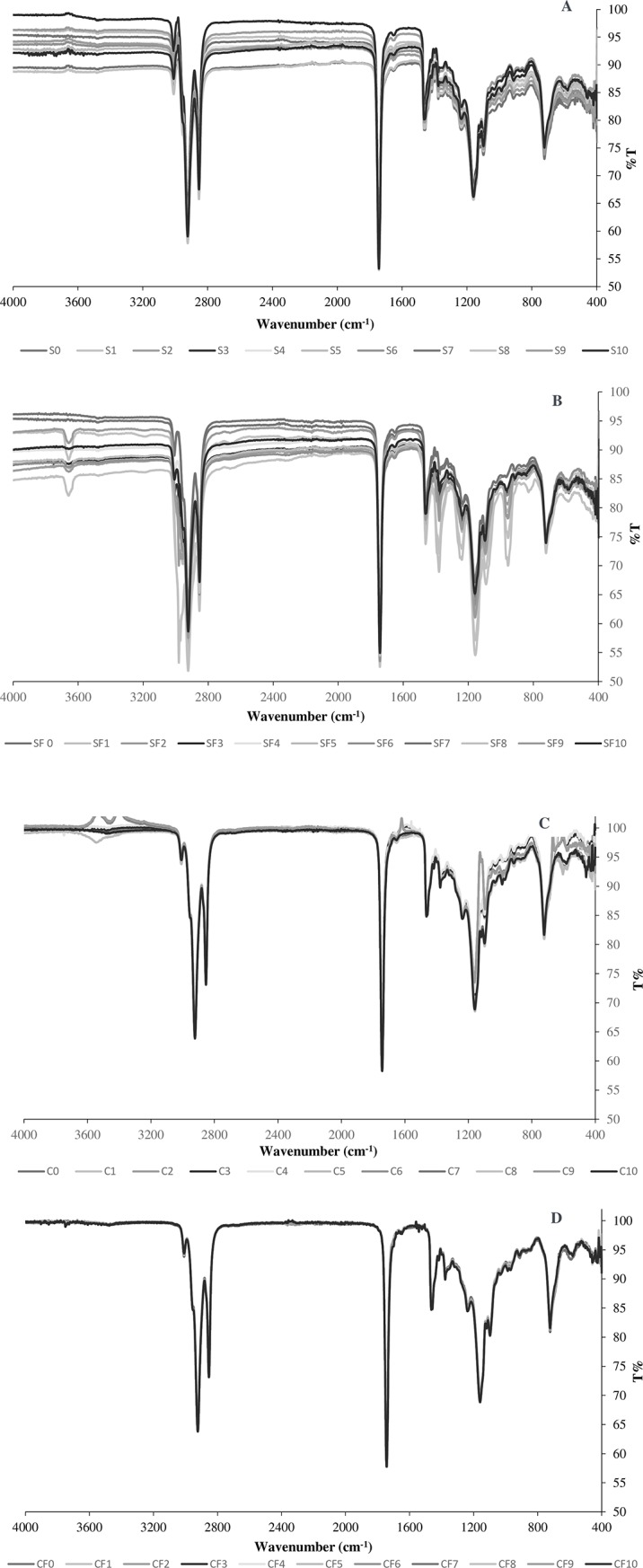
FTIR spectra of (a) sunflower oil (S) samples that were heated for 1 hour/day, (b) sunflower oil (SF) samples that were used for fries frying for 1 hour/day, (c) corn oil (C) samples that were heated for 1 hour/day, (d) corn oil (CF) samples that were used for fries frying for 1 hour/day.

Fig 5 depicts the FTIR spectra recorded for 10 samples of the sunflower oil and corn oil that were acquired after each heating process at 180°C for 1 hour per day (S1 to S10 for sunflower and C1 to C10 for corn oil; the number beside the letter represents the number of days of heating). A close interpretation of the spectra revealed that there are some changes in the transmittance percent of some bands as well as some slight shifts in the exact position of the bands. This change proposes that the oil composition has effects on the exact position of the bands and shifts in the bands when the fatty acid composition is changed. Table 3 shows the main functional groups and their corresponding wavenumbers observed for the studied oils.

**Table 3:** The main functional groups observed on the studied oils by FTIR spectroscopy and their corresponding wavenumbers.

Wavelength (cm^-1^)	Functional group/bands	Reference
3007–3009	C-H symmetric stretching vibration of the *cis* double bonds, = CH	[49]
Shoulder at 2960–1980	C-H asymmetric stretching vibration of aliphatic CH_3_ groups	[50]
2853–2854 and 2922–2923	C–H asymmetric stretching vibration of aliphatic CH_2_ group	[49, 51]
~1743	ester carbonyl stretching vibration of the functional groups of the triglycerides, C = O	[49]
weak shoulder at 1700	free fatty acid carbonyl group stretching vibration, C = O	[50]
1458 and 1463	C–H bending vibration of CH_2_ and CH_3_ aliphatic groups	[50]
1377	C–H bonds bending symmetric vibration of CH_2_ groups	[49]
1237, 1159, 1118 and 1097	C–O stretching vibration of the ester groups	[51]
967	out-of-plane bending vibration of *trans–*HC = CH− group of di-substituted olefins	[51]
722	overlapping of CH_2_ rocking vibration and the out-of-plane vibration of *cis–*HC = CH− group of disubstituted olefins	[49]

Corresponding to the previous results of Poiana et al. [44], which demonstrates that there was not much difference between the features and characteristics of the oil, other than slight shifts which are affected by the proportion of the composition of the oil. Poiana et al. [44] observed that at the band around 3006 cm^-1^ had a slight difference in the maximum absorption position between the olive oil (3005 cm^-1^), olive oil that was mixed with soybean oil and the pure soybean oil (3009 cm^-1^). It was explained by Vlachos et al. [49], that the changes in the exact position of the maximum absorption of the infrared at 3006 cm^-1^ band was related to the unsaturation degree of the vegetable oils.

**Fig 6:**
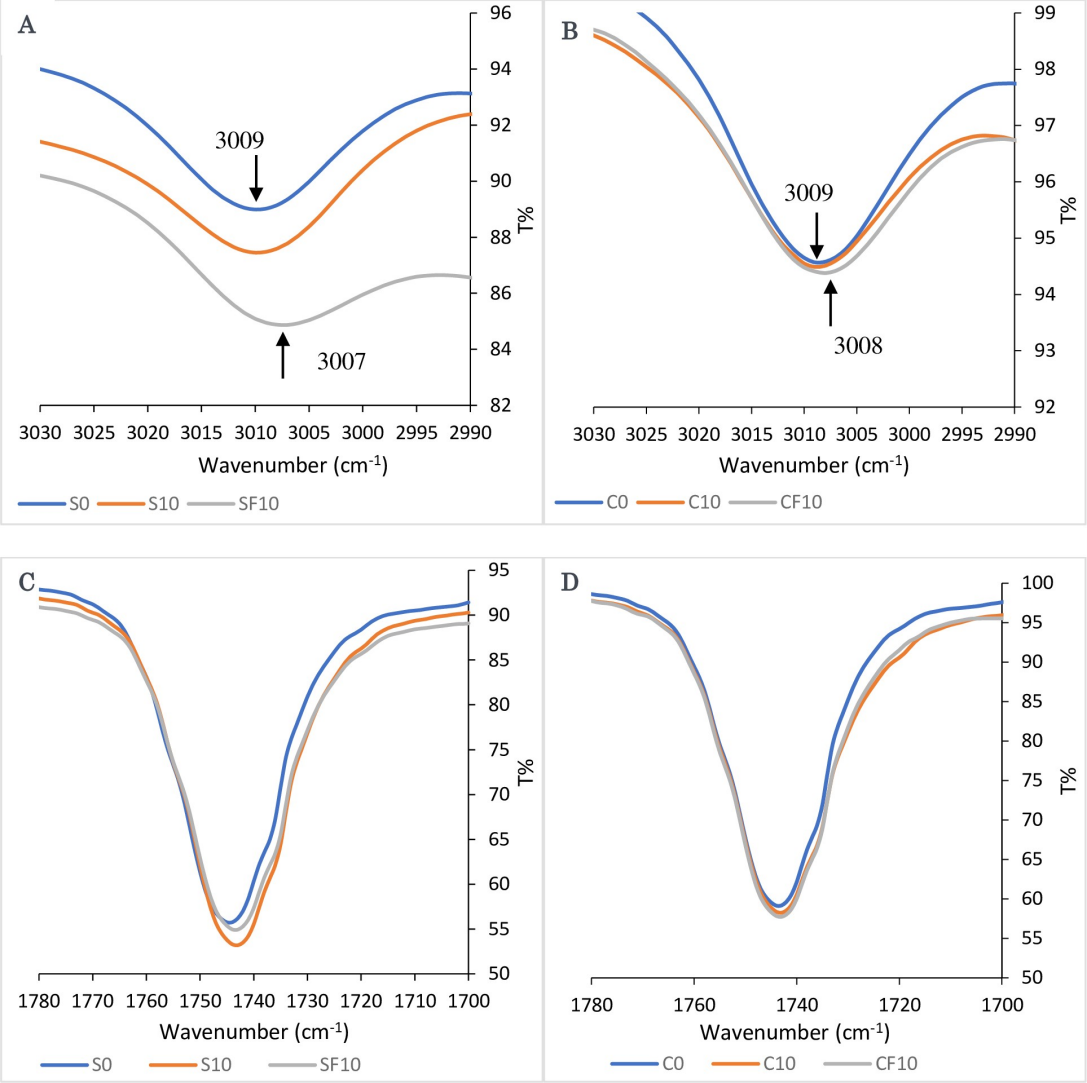
FTIR spectra of the 3006 cm^−1^ band shifts of (a) sunflower oil samples, (b) corn oil samples in response to heating and frying and the changes in the C = O region of infrared spectra for (c) sunflower oil samples, (d) corn oil samples.

Here, Fig 6 shows a notable difference around the 3006 cm^-1^ band, where the pure sunflower oil and corn oil along with the 10^th^ sample of the heated oil had a minimum transmission at 3009 cm^-1^. The minimum transmissions of 10^th^ sample of the corn and sunflower oils that were used for French fries frying were 3008 and 3007 cm^-1^, respectively. These findings suggest that the heating process has no major effect on the unsaturation degree of the oil. But on the other hand, the frying process has an effect on the unsaturation degree, where the oil gets more unsaturated which means it loses more hydrogen atoms. This revealed that the oil gets oxidized which is in agreement with the previously reported results by Poiana et al. [44], and Vlachos et al. [49]. From the results, it could be observed that the main reason behind the oxidation of oil was the added moisture from the food in combination with the heating process, but not only heating.

Another finding that can be noticed from Fig 6 is that the height of the bands around 3006 cm^-1^ is significantly different, it was revealed by Poiana et al. [44], in their experiment that this height difference was also an evidence of the change of unsaturation degree. In their results, the band height increments along with the degree of adulteration of the olive oil. Thus, as a measure of the changes in the degree of unsaturation in response to oil oxidation, the change in the transmittance at 3006 cm^–1^ was taken into consideration. Where the pure sunflower and corn oils show the highest transmittance percent, in another word, the lowest absorption, which means the less unsaturation degree, followed by the heated sunflower oil and corn oil, and the lowest transmittance percent was for the sunflower and corn oil that was used for fries frying which means it has the highest unsaturation degree and the highest oxidation level.

Fig 6 shows the spectral changes recorded in the C = O region for the sunflower oil and corn oil. The 1690–1760 cm^−1^ represented the vibration of the carboxylic acid of the triglycerides, which includes ester, ketone, and aldehyde. Two bands made up the main carbonyl band at 1743 cm^−1^ a sharp constituent appears and the broad constituent at 1728 cm^−1^ [52]. The results showed that the peak of the C = O region gets wider and slightly change the exact position of the band recorded at 1743 cm^–1^ for the used oil samples. It was reported by Poiana et al. [44], Vlachos et al. [49], Moharam and Abbas [50], and Guillen at al. [53], that the changes in the band at 1743 cm^−1^ in the FTIR spectra could be as a result of the formation of saturated aldehydes and the hydroperoxides decomposition or other secondary oxidation products that cause absorption at the band 1728 cm^−1^ like alcohol, ketones, acids, and esters, which may overlay with the stretching vibration band at 1743 cm^−1^ of the ester carbonyl functional group of the triglycerides. In our findings, it can be clearly observed that the heated oil (S10 and C10) and the frying oil (SF10 and CF10) samples showed a wider band at 1743 cm^-1^ and less transmittance percent, which is an evidence that an oxidation process and a thermal degradation has taken place. Furthermore, when aldehyde and ketone compounds form carbonyl compounds in thermo-oxidation conditions, this will affect the minimum transmission of the band at 1700 to 1726 cm^-1^, and increase the broadening of the band with a decline in the frequency to 1743 cm^-1^ [44]. The degree of oxidation deviations that was monitored using FTIR was affected by the duration of heat exposure [44].

**Fig 7:**
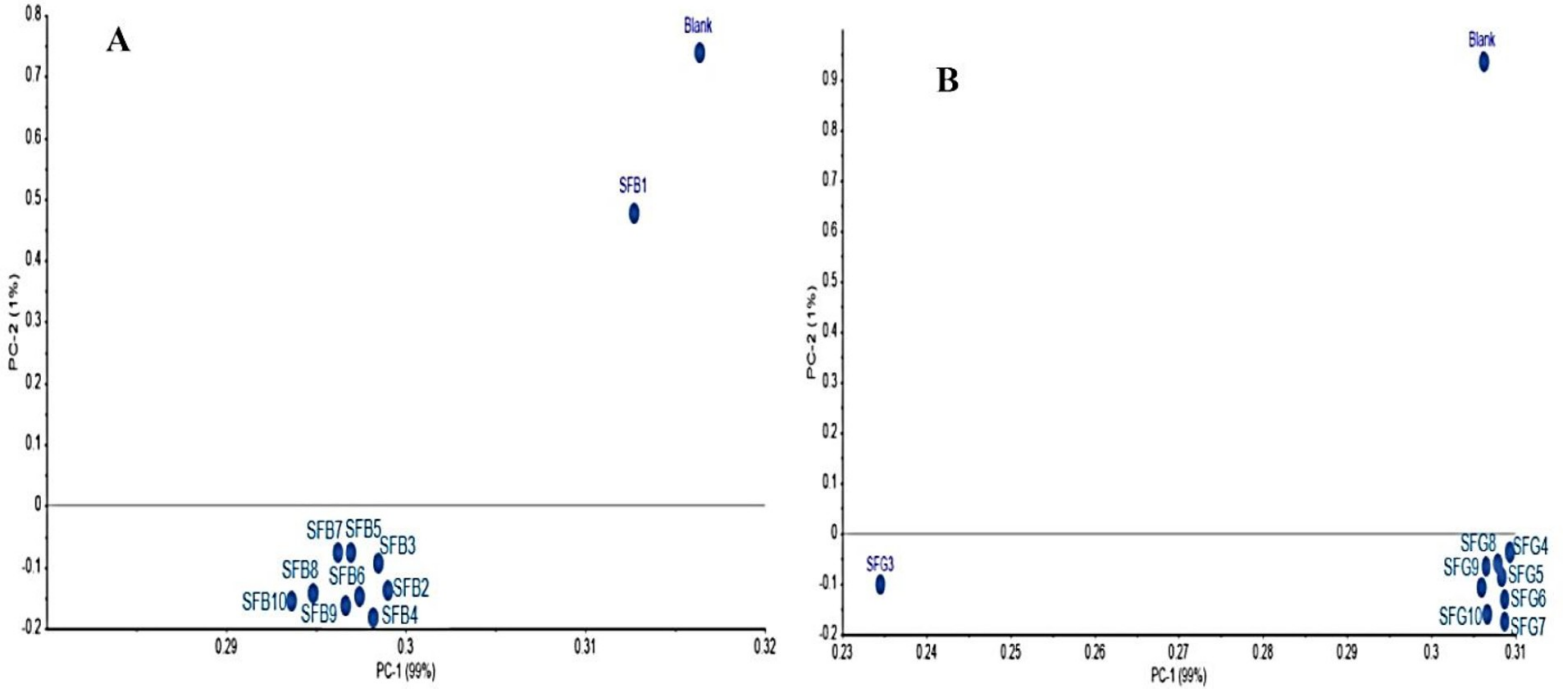
PCA done for FTIR spectra of activated carbon prepared from black (SFB) and green (SFG) olive stone after treatment of sunflower oil.

**3.2.2.2. FTIR analysis–used sunflower and corn oils–the batch adsorption using the green OSAC and black OSAC.** To study the efficiency of the green OSAC and black OSAC as adsorbents for removing the oxidation products from the used sunflower and corn oils (obtained from the heating and frying processes), the FTIR analyses were carried out in addition to the PCA analysis. The PCA for the FITR spectral changes showed that significant variations in the green OSAC and black OSAC occurred after adsorption. The PCA clustered FTIR spectra after adsorption were together and away from the blank spectrum i.e. the spectrum of unused oil (Fig 7). However, a closer interpretation of the spectra revealed that there are some changes in the transmittance percent of some bands as well as some slight shifts in the exact position of the bands before and after batch adsorption treatments with the green OSAC and black OSAC.

**Fig 8:**
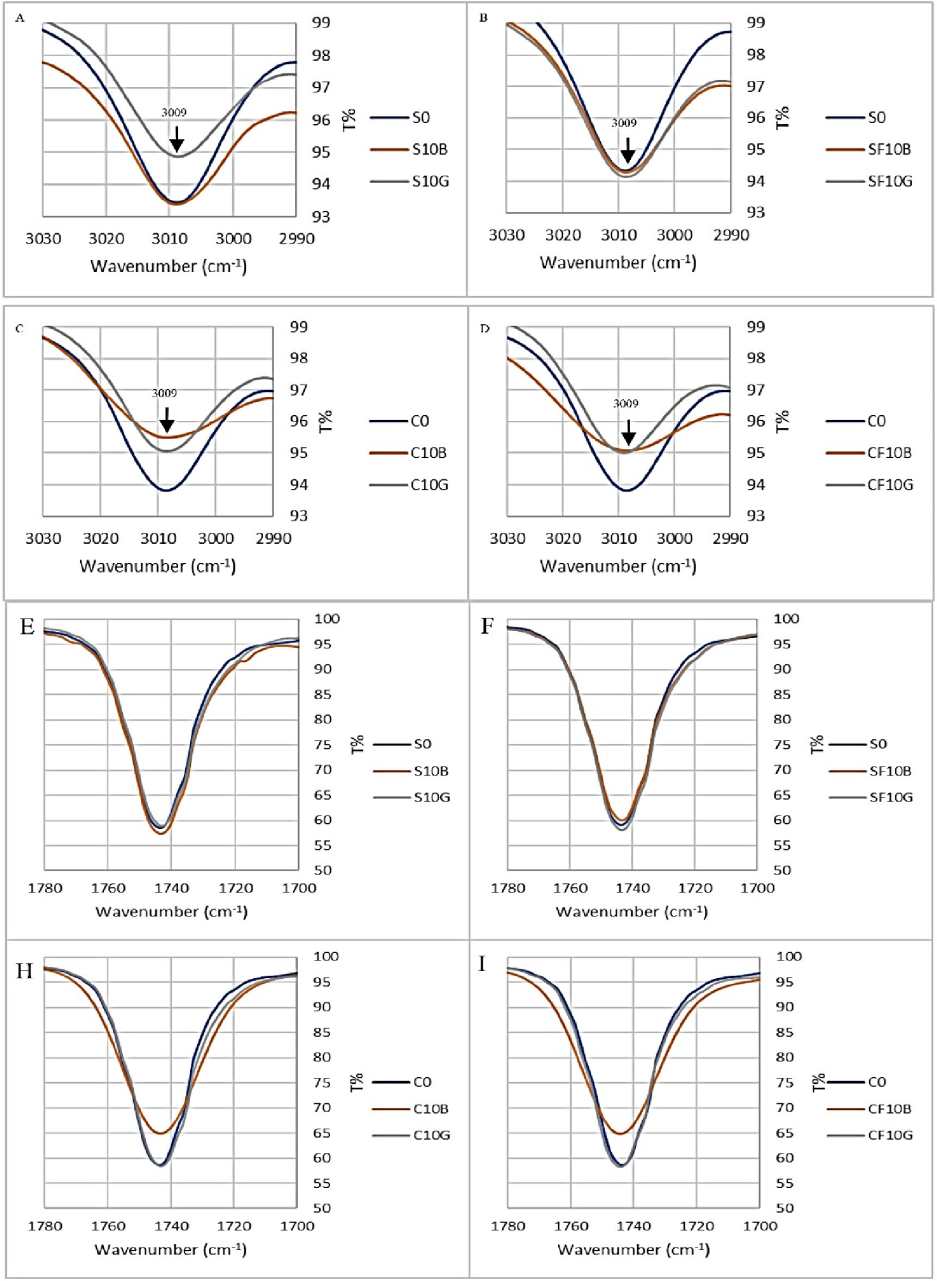
FTIR spectra of the 3006 cm^−1^ band (C-H symmetric stretching vibration of the cis double bonds, = CH) of sunflower oil and corn oil samples after treating with black OSAC and green OSAC (A) heated sunflower oil (B) frying sunflower oil (C) heated corn oil (D) frying corn oil, and FTIR spectra of the ~1743 cm^–1^ (ester carbonyl stretching vibration, C = O) (E) heated sunflower oil (F) frying sunflower oil (H) heated corn oil (I) frying corn oil.

As discussed before and explained by Vlachos et al. [49], that the changes in the exact position of the maximum absorption of the infrared at the band 3006 cm^-1^ are related to the unsaturation degree of the vegetable oils. From the previous results, it was shown that the minimum transmission at the 3006 cm^-1^ band for both pure sunflower and corn oil was at 3009 cm^-1^. After the oil oxidation, there was a slight shift in the exact position of the minimum transmission at the band. After treating the samples with the green OSAC and black OSAC, it can be observed from Fig 8 that all the minimum transmission position of all the samples was at 3009 cm^-1^, which is the same position of the pure oil minimum transmission. It can be concluded that the green OSAC and black OSAC were efficient in reducing the unsaturation degree which was related to the reduction of oxidation degree.

As mentioned before that the changes in the band at 1743 cm^−1^ in the infrared spectra could be due to the formation of saturated aldehydes and the hydroperoxides decomposition or other secondary oxidation products. Furthermore, when the oil was used (being oxidized), the results showed that the peak (C = O region) gets wider, less transmission percent and slightly changed the exact position of the band recorded at 1743 cm^–1^. Fig 8 shows the changes that happened to the peak at band 1743 cm^-1^ after batch adsorption treatment with the green OSAC and black OSAC. It can be clearly observed that the peaks get closer to the pure (fresh) oil peaks (namely S0 and C0) and the minimum transmission percent had increased in some of the peaks. This is evidence that the green OSAC and black OSAC were efficient to remove the oxidation products, as it shows a reduction in the C = O amount in the oil.

#### 3.2.3.Ultra-Performance Liquid Chromatography (UPLC) experiment

The UPLC was used to quantify the level of the polar compounds in the oxidized sunflower and corn oils. This was used as an indicator of the oxidation level. It was a very challenging task to analyze the oils using the UPLC as the separation process was complex. Vegetable oils are a complex mixture that has many components that are very non-polar including fatty acids, triglycerides, waxes, sterols, hydrocarbons, vitamins, and others. For this reason, the sunflower and corn oil samples were solid-phase extracted using silica gel cartridges with dichloromethane to get rid of the non-polar components and extract the polar compound only after nitrogen evaporation of the dichloromethane. In a review that was done by Cert et al. [54], it was mentioned that better results of the polar compounds composition analysis were achieved, when the samples were solid-phase extracted using silica gel cartridges, unlike when a column chromatography was used.

**Fig 9:**
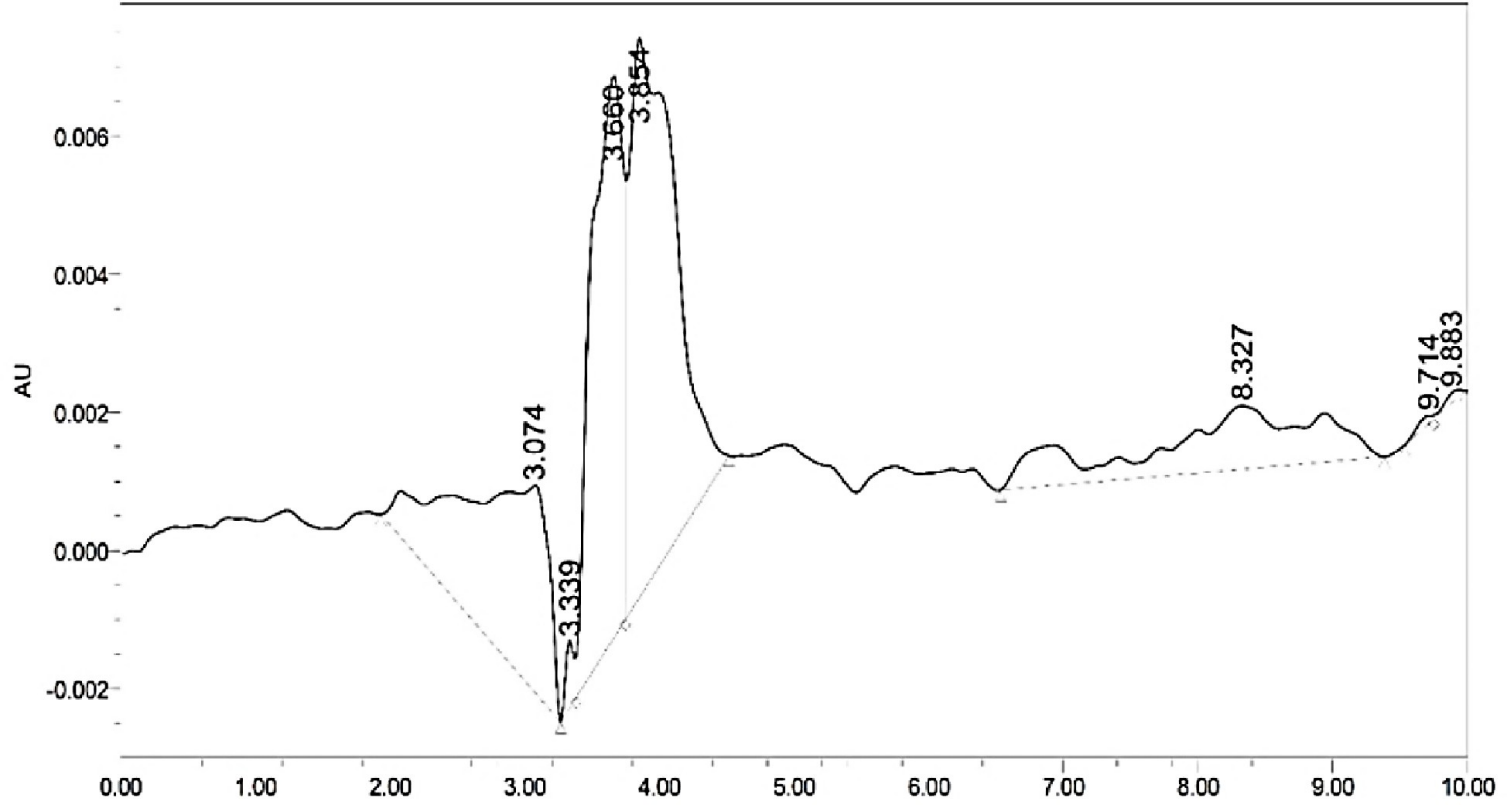
Detailed UPLC chromatogram of pure sunflower oil (blank).

Fig 9 shows the UPLC chromatogram of the fresh sunflower oil after the solid-phase extraction. Here, the peak of the polar compound was used as an alternative measurement for the amount of oxidation. In a study that was done by Velasco et al. [55], where they compared the common HPLC device results with other analytical techniques results in the determination of oxidation products in sunflower oil, the HPLC method was good enough in the determination of the quantities of the abundant oxidized lipids with good linearity, sensitivity, precision and accuracy of the results. It was investigated by Amelio et al. [3], in their experiment to separate the wax esters from olive oil that the HPLC results are double to triple time more precise than method No. 183/93 in the Economic Community Regulation.

Fig 9 shows that almost all major peaks appeared around 4^th^ minute. In an experiment that was done by Xiu-Qin et al. [56], HPLC was used to analyze antioxidants and preservations in vegetable oil. In their experiment, they used a Zorbax C18 column with a mobile phase of acetonitrile and water which is the same as what was used in our experiment. Where in our experiment, the extracted oil sample was injected in the UPLC. Using column Zorbax C18 with 50:50 v:v Acetonitrile: water at flow rate 1 mL/min, detected peaks at 254 nm with a UV detector. In their results, at minute 4.44 a peak appeared that represent propyl gallate (PG), which is a form of an ester. In our results, to calculate the amount of oxidation, the area of the major peaks around the 4^th^ minute was summed up from each sample chromatogram, and then the blank sample peaks area was subtracted from the results to get the net area. It was observed that the net area of the peaks was constant until the 3^rd^ hour of heating, then it became roughly stable in which it was fluctuating around a small limited range. In the results, the area of the peaks represents the increment in the oxidation level. The heating process influenced the increase in oxidation level until the 3^rd^ hour, unlike the frying process that had an effect until the 6^th^ hour. It can be noticed that the frying process has two times the effect on the oxidation level than the heating process. In addition, the fluctuation of the peak net area can be justified by the remaining of the dichloromethane that was used in the solid phase extraction experiment. The nitrogen evaporation was done manually, which means that a different amount of dichloromethane residue was still in the samples, which obviously give an uneven effect on the results and cause some fluctuation.

**Fig 10:**
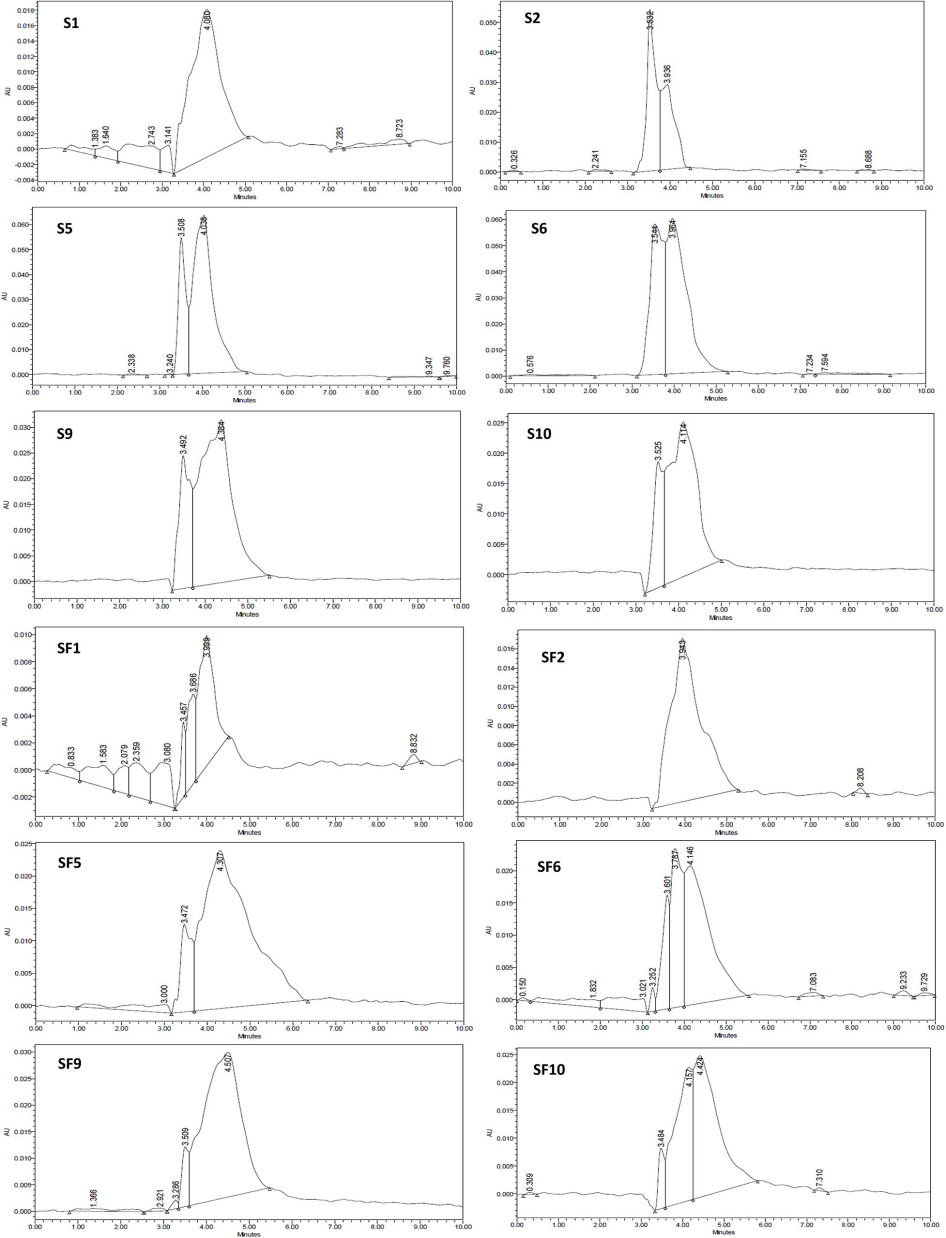
UPLC chromatograms for all of the samples, each heating or frying hour.

To sum up, it can be noticed that the UPLC results support the FTIR results. Both methods showed that heat and frying affected the content of the polar compounds of the oil and the oxidation level. The frying process had more effect that reaches to double the effect of the heating process, this can be due to many reasons like the food moisture, proteins and carbohydrate from food, small food particles leftover in the oil, and the air insertion with the foaming reaction when the frozen fries was dipped in the hot oil (Fig 10).

### 3.3. Mechanisms of oxidation products’ adsorption by OSAC

Knowing the mechanisms of oxidation product adsorption on the surface of the green OSAC and black OSAC is important for the removal process of the oxidation product from the used cooking oil. The oxidation products that appear in the used cooking oil have different chemical and physical characteristics that result in different interactions’ ways with the different adsorbents’ types. The main impetus for adsorption results from either the solubility or the special properties of the solute concerning the solvent, or the specific attraction force of the solute to the adsorbent [57]. The way of adsorption can be mainly an electrical attraction (van der Waals) or the chemical nature of the components. Understanding the adsorbent surface chemistry is mandatory to know how it is affecting the adsorption process and to select the most suitable adsorbent for each pollutant. To know the ability of an adsorbent to adsorb a specific material and its adsorptive capacity, it needs further understanding of the adsorbent surface chemistry in addition to the textural porous structure of the adsorbent. This is because the adsorption capacity can be influenced by the surface attraction force if there are unpaired electrons on the surface of the adsorbent or incomplete saturated valences or some functional groups, especially when more quantities of adsorbate are taken up onto the adsorbent by means of chemisorption.

Activated carbons are broadly utilized as adsorbents, in which it was used in many studies for the adsorption of pollutant and oxidants from diesel and used lubricants. They exemplify very adaptable adsorbents of industrial significance and are broadly utilized in numerous applications which concern basically with the elimination of unfortunate species from fluids or gases as it has a large surface area with many surface functional groups that make it able to adsorb a variety of pollutant from a wide range of mediums. An important role in the applications of activated carbon is done by the heteroatoms on its surface. The heteroatoms of permeable carbon surface primarily contain oxygen, nitrogen, hydrogen, halogen, and so on, which clung to the edges of the carbon layers and control the surface of the activated carbon [58]. Moreover, the functional groups that contain oxygen and known as surface oxides were the most common species within the heteroatoms and the widely formed in the surface of the activated carbon, which in turn, plays an important role in the adsorption performance of activated carbon as well as in its conversion systems and catalytic reactions [59]. Many other functional groups can result from the heteroatoms mainly carboxyl, lactonic, carbonyl, quinone and phenolic. Those surface functional groups have multiple shapes of a crevice, voids, and cavities that attach to aromatic sheets and strips (Fig 10) [60]. Moreover, the surface chemical functional group of the adsorbent could be acidic or alkaline. This affects the pollutants’ adsorption process in many ways. However, the adsorption process conditions like concentration, time, and temperature, could affect the solution pH [61].

Activated carbon can have acidic and the basic pH, and the surface functional groups make electrical charge properties, which strongly affect the adsorption of the ionic and polar compounds. The solution pH can show basic properties when there is a basic surface oxide that exists, where the low oxygen content with the carbon shows anion exchange behavior with a basic pH. On the other hand, when the surface oxides are acidic, they show acidic properties in the solution pH value, where high oxygen content with carbon shows cation exchange behavior with acidic pH [60]. A counterbalance of the activated carbon surface charge should be maintained in the solution phase to stay electroneutrality, and since the oxidation products in the used cooking oil are acidic compounds, so, basic active sites are needed for effective adsorption, where the basic sites are related to the surface oxygen [57].

**Fig 11:**
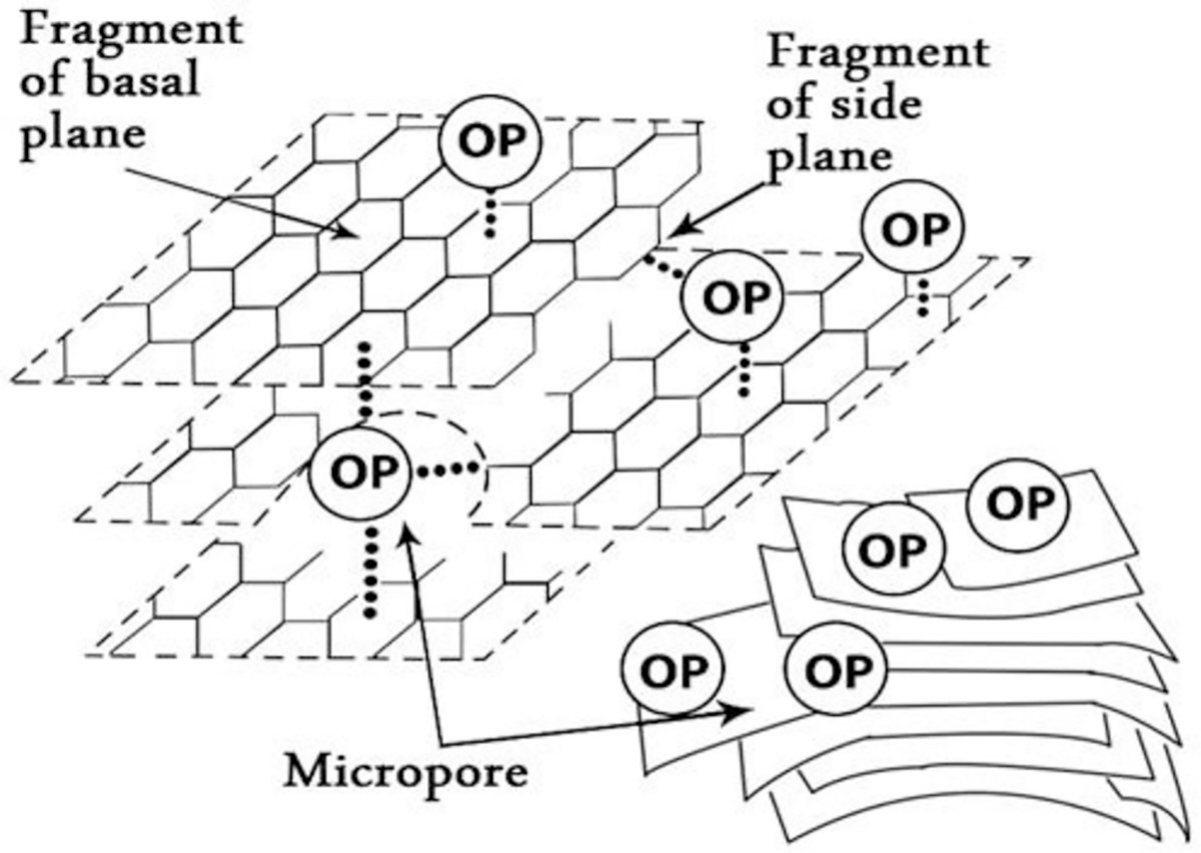
Types of adsorption sites that occur on activated carbon: Basal plane, edge plane, and micropores.

The adsorption process starts in the external surface of the OSAC where the surface functional group contribute to the adsorption. Then the adsorption process happens in the whole surface where the adsorbed oxidation products are distributed on the surface of the OSAC. The oxidation products that are adsorbed on the surface of the OSAC forms π- complexes with the C = C parts of the OSAC system [57]. Fig 11 shows the types of adsorption sites that happen to the OSAC when it adsorbs oxidation products. Furthermore, FTIR is used to monitor the interaction between the active group of the adsorbent surface and the adsorbate, as it is important for the adsorption process [61]. The FTIR technique was used in our study to monitor the contribution of the OSAC functional group in the adsorption of the oxidation products. Between the adsorbent and the adsorbate, a weak bond can be formed (π–π complexation) [62]. The density of the π-electrons in the adsorbate influence the degree of π- complexation in the adsorption reaction between the adsorbent and adsorbate. The molecules that contain more than two double bonds, triple bonds, and polynuclear aromatic, very strong bonds can be formed with them. The adsorbents can be modified to produce the needed strength of bond by selecting the suitable cation [57]. In an experiment that was done by Al-Ghouti and Al-Degs [57], activated carbon was efficient in adsorbing oxygen, nitrogen, and sulfur products in waste lubricants more than the raw diatomite, which might be associated to hydrogen bonds; competition for adsorption sites; electrostatic field strength and intraparticle diffusion of molecules; and hydrophobic interaction.

### 3.4. Cost efficiency of using olive stone as an adsorbent

As mentioned previously that olive stone contains low-fat content, allowing them to be utilized in olive mills for the production of oil with low-quality. Olive stone remaining from the olive mills can be utilized as an energy resource after drying. Technical and economic drawbacks from the direct combustion of olive stone due to the limited fraction of fixed carbon, high moisture content as well as the low energy density [63]. Furthermore, olive stone can be applied in multiple industrial applications as shown in Table 4.

**Table 4:** Various applications of olive stone and their application sector.

Application	Application sector	References
Combustion	All industries, residential and commercial	[64, 65]
Activated carbon production	Food, chemical, petroleum, nuclear, mining, and pharmacological industry	[66–69]
Bio-oil production	Various industrial fields	[70]
Furfural production	Used as a solvent or as a base for synthesizing its derived solvent	[71, 72]
Used as plastic-filled	Plastic and construction	[73, 74]
Abrasive	Various industrial fields like cleaning	[75]
Cosmetic	Cosmetic sectors	[76]
Biosorbent	Metallurgy and food	[76]
Resins	electrochemical	[77,78]
Fractionation	Food, cosmetic, pharmaceutical and alcohol	[76]

Using olive stone as an adsorbent for the treatment of various pollutants is more cost-efficient than using it as part of biomass briquettes for woodstove use, for example due to the high cost of the binders used in briquetting. Furthermore, there is no enough information regarding the implementation of torrefaction combined with briquetting as an efficient management treatment to reduce the amount of organic waste [79]. On the other hand, using olive stone as an adsorbent is flexible, simple, and inexpensive approach due to the zero economic value of olive stone as they are considered as waste leading to a reduction in the process cost, as well as having the ability to be regenerated and aiding in solving olive stone disposal problems. Furthermore, it was concluded by kyzas and Kostoglou [80], who investigated the techno-economic analysis of various adsorbents and compared the estimated cost for the adsorbent production from non-modified agricultural waste like olive stone and activated carbon and found that producing activated carbon from non-agricultural sources costs at least 4 times the production cost of using agricultural waste due to the increased electricity demand, making adsorbents from agricultural wastes such as olive stone more cost-efficient.

## 4. Conclusion

The novelty of this work relies on the use of the green OSAC and black OSAC for the treatment of used sunflower and corn cooking oil. Elemental and morphological analysis showed that the OSAC had an irregular porous structure with large, deep well-developed cavities, with similar CHNS percent to CAC. The FTIR main oxidation peaks showed a decrease in the oxidation level of the used oil after treatment. The results of FTIR-PCA showed that significant variations in the oxidation level of sunflower and corn oil occurred after use for consecutive 10 days for heating and frying French fries. There are various applications for olive stone in different sectors, however, using olive stone as an adsorbent is more beneficial as it helps in solving the disposing problem faced by the main producing countries since it is produced in tremendous quantities. It can be concluded that OSAC can be promising, efficient, economically effective, and environmentally friendly alternative adsorbent for oil treatment applications. This study is the first step in applying the use of olive stone as an adsorbent for oil in lab-scale and our prospects are to help in applying it on a pilot scale.

## Abbreviation

OSAC: Olive stone activated carbon
S: Sunflower oil
C: Corn oil
S0,S1,S2,….S10: Sunflower oil–The number beside the letter represents the number of days of heating
C0,C1,C2,….C10: Corn oil–The number beside the letter represents the number of days of heating
S0: Fresh sunflower oil
C0: Fresh corn oil
SF: Sunflower oil after French fries frying
CF: Corn oil after French fries frying
SF0,SF1,SF2,….SF10: Sunflower oil after French fries frying–The number beside the letter represents the number of days of heating
CF0,CF1,CF2,….CF10: Corn oil after French fries frying–The number beside the letter represents the number of days of heating
SFB: Sunflower oil after French fries frying–black OSAC
SFG: Corn oil after French fries frying–green OSAC
FTIR: Fourier transfer infrared
SEM: Scanning electron microscopy
PCA: Principle component analysis
SPE: Solid-phase extraction
UPLC: Ultra performance liquid chromatography
HPLC: High-performance liquid chromatography
CHNS: Carbon, hydrogen, nitrogen, sulfur analysis
OP: Oxidation products
